# Cardiac Alternans Occurs through the Synergy of Voltage- and Calcium-Dependent Mechanisms

**DOI:** 10.3390/membranes11100794

**Published:** 2021-10-18

**Authors:** Minh Tuan Hoang-Trong, Aman Ullah, William Jonathan Lederer, Mohsin Saleet Jafri

**Affiliations:** 1 Krasnow Institute for Advanced Study and School of Systems Biology, George Mason University, Fairfax, VA 22030, USA; hoangtrongminhtuan@gmail.com (M.T.H.-T.); aullah3@gmu.edu (A.U.); 2 Center for Biomedical Engineering and Technology, University of Maryland School of Medicine, Baltimore, MD 21201, USA; JLederer@som.umaryland.edu

**Keywords:** heart, arrhythmia, computational model

## Abstract

Cardiac alternans is characterized by alternating weak and strong beats of the heart. This signaling at the cellular level may appear as alternating long and short action potentials (APs) that occur in synchrony with alternating large and small calcium transients, respectively. Previous studies have suggested that alternans manifests itself through either a voltage dependent mechanism based upon action potential restitution or as a calcium dependent mechanism based on refractoriness of calcium release. We use a novel model of cardiac excitation-contraction (EC) coupling in the rat ventricular myocyte that includes 20,000 calcium release units (CRU) each with 49 ryanodine receptors (RyR2s) and 7 L-type calcium channels that are all stochastically gated. The model suggests that at the cellular level in the case of alternans produced by rapid pacing, the mechanism requires a synergy of voltage- and calcium-dependent mechanisms. The rapid pacing reduces AP duration and magnitude reducing the number of L-type calcium channels activating individual CRUs during each AP and thus increases the population of CRUs that can be recruited stochastically. Elevated myoplasmic and sarcoplasmic reticulum (SR) calcium, [Ca^2+^]_myo_ and [Ca^2+^]_SR_ respectively, increases ryanodine receptor open probability (P_o_) according to our model used in this simulation and this increased the probability of activating additional CRUs. A CRU that opens in one beat is less likely to open the subsequent beat due to refractoriness caused by incomplete refilling of the junctional sarcoplasmic reticulum (jSR). Furthermore, the model includes estimates of changes in Na^+^ fluxes and [Na^+^]_i_ and thus provides insight into how changes in electrical activity, [Na^+^]_i_ and sodium-calcium exchanger activity can modulate alternans. The model thus tracks critical elements that can account for rate-dependent changes in [Na^+^]_i_ and [Ca^2+^]_myo_ and how they contribute to the generation of Ca^2+^ signaling alternans in the heart.

## 1. Introduction

The alternating strong and weak beats in the left ventricle are known as pulsus alternans or mechanical alternans which was first described in the 19th century by Traube [1]. Another type is electrical alternans (or T-wave alternans) which describe the beat-to-beat variation in direction, amplitude, and duration of any components in an ECG waveform [2]. The two are distinguished, yet they may both coexist [3,4].

Pulsus alternans is associated with different pathophysiological conditions, e.g., aortic stenosis, tachycardia, ischemia, acidosis and hypertrophic cardiomyopathy [5]. Other tested conditions that may lead to cellular or subcellular alternans include, but are not limited to: decrease RyR2 open probability (P_o_) to increase the variability of Ca^2+^ transient between the different areas of the cell, metabolic deficiencies, e.g., acidosis, and/or abnormal calcium handling [4,6,7]. Pulsus alternans may lead to pulseless activity, i.e., when there is an electrical activity, but the heart either does not contract, or the contraction is not strong enough to produce a sufficient cardiac output to generate a pulse [8].

It has been suggested that a voltage-dependent mechanism underlies cardiac alternans. Under this hypothesis, action potential restitution is the underlying cause of cardiac alternans [9,10,11,12,13]. With the shorter diastolic interval with faster pacing rates, the sarcolemmal ion channels do not completely recover from one beat to the next. Therefore, after a large/long action potential, since ion channels have not fully recovered, they are not available to participate in the next action potential resulting in a small/short action potential. In these studies, a steep restitution curve is the primary requirement, and pathological conditions or experimental manipulations can increase the slope of this restitution curve making alternans more likely.

Other studies have suggested, that modified intracellular calcium cycling plays a role in occurrence of mechanical and electrical alternans [14,15,16,17,18,19]. In fact, many studies have found that it is possible to have alternans in calcium release without requiring action potential alternans [18,20,21]. Calcium alternans is a beat-to-beat variation in intracellular Ca^2+^ transient amplitude. Typically, calcium alternans occurs at high heart rates, yet the frequency threshold varies by different conditions such as ischemia or ionic disturbances that disturb the bidirectional coupling between the membrane potential and intracellular calcium. This has been identified as a potential precursor to the dangerous reentrant arrhythmias and SCD; yet the mechanism is not well understood [22]. Current computational models are unable to recreate this phenomenon; unless certain modifications to the ionic currents were made [9,23].

Newer studies have suggested that both mechanisms are possible under different conditions [24,25]. Those studies used computational models to suggest specific model configuration that can produce alternans by either a calcium-dependent or voltage-dependent mechanism. They then used rapid pacing in guinea-pig ventricular myocyte and observed calcium-dependent alternans under conditions of control of action potential alternans. They suggested that in these myocytes, a voltage-driven mechanism was possible due to the steep action potential duration restitution curves.

In this study we use our local-control model of the rat ventricular myocyte to study the interplay of calcium-dependent and voltage-dependent mechanisms to give rise to rapid pacing induced alternans. Through dissection of calcium dynamics and sarcolemmal ion channel activity at the release site level we demonstrate that for alternans brought about by increased pacing rate, there is a synergy between these two mechanisms. The modeling suggests that restitution of the action potential due to incomplete recovery of the Na^+^ channels that sets the stage for alternans by a calcium dependent mechanism. Here the small action potential reduces the number of L-type calcium channels that are activated creating the substrate for stochastic activation of the ryanodine receptors that is favored by elevated myoplasmic calcium.

## 2. Materials and Methods

### 2.1. The Model

**Figure 1:**
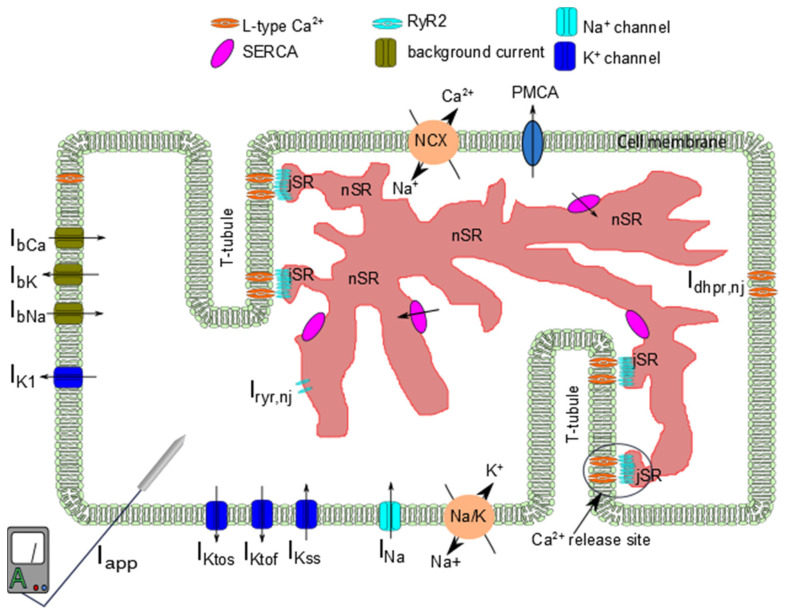
Schematic diagram of a ventricular cell that shows only 2 T-tubule branches with 4 or the 20,000 Ca^2+^ release units. Abbreviations: jSR—junctional sarcoplasmic reticulum; nSR—network sarcoplasmic reticulum; ryr—ryanodine receptor; T-tubule—transverse tubule; NCX—sodium-calcium exchanger; Na/K—sodium-potassium ATP ase; PMCA—plasmalemmal calcium ATPase; SERCA—sarcoplasmic and endoplasmic reticulum calcium ATPase; I_Na_—sodium current; I_Kss_—steady-state (sustained) potassium current; I_Ktof_—fast transient outward potassium current; I_tos_—slow transient outward potassium current; I_K1_—inward rectifier potassium current; I_bCa_ = background calcium current; I_bK_—background potassium current; I_bNa_—background sodium current.

In order to study the mechanisms of alternans, we developed a novel local-control model for excitation-contraction coupling in the rat ventricular myocytes with 20,000 Ca^2+^ release sites that improves upon our previous work [26,27,28] (Figure 1). Notable improvements of this model over previous work include the following: (1) explicit buffer dynamics in the subspace, (2) an improved L-type calcium channel that incorporate calcium-bound calmodulin dependent inactivation, (3) updated formulation of Ito, and (4) updated parameters based on newer experimental data from rat ventricular myocytes [29]. In addition, by using our patented Ultra-fast Monte Carlo Method and GPU technology, it now allows us to do larger scale simulations that provide insights into calcium dynamics. The model equations are detailed in Appendix B.

#### 2.1.1. Calcium Release Site (CRU)

A calcium release site is formed by the dyadic subspace and contains a cluster of 49 RyR2 channels and 7 LCCs channels. At each calcium release site, dynamic calcium buffering is implemented for three different endogenous buffers: calmodulin (CaM), sarcolemmal (SL) buffer and sarcoplasmic reticulum (SR) buffer rather than using the rapid buffering approximation or fixed buffering [27,30].

#### 2.1.2. Ryanodine Receptor Type-2 Model

The 2-state ryanodine receptor model incorporates cytosolic calcium-dependent and luminal calcium dependent gating as described previously with only a small modification to the luminal dependence function to match spark characteristics [27]. The states are with the transition rate $k_{ryr}^{+}$ being increased by subspace Ca^2+^ (${\left[{\mathrm{Ca}}^{2+}\right]}_{ds}^{i}$) and junctional SR Ca^2+^ (${\left[{\mathrm{Ca}}^{2+}\right]}_{jsr}^{i}$) and $k_{ryr}^{+}$ being a constant. The RyR2s are arranges in a cluster and display coupled gating as using our previous formulation [27].

(1) $$C\begin{array}{c} \overset{k_{ryr}^{+}}{\to }\\ \underset{k_{ryr}^{-}}{\leftarrow } \end{array}O$$

#### 2.1.3. L-Type Ca^2+^ Channel Model

**Figure 2:**
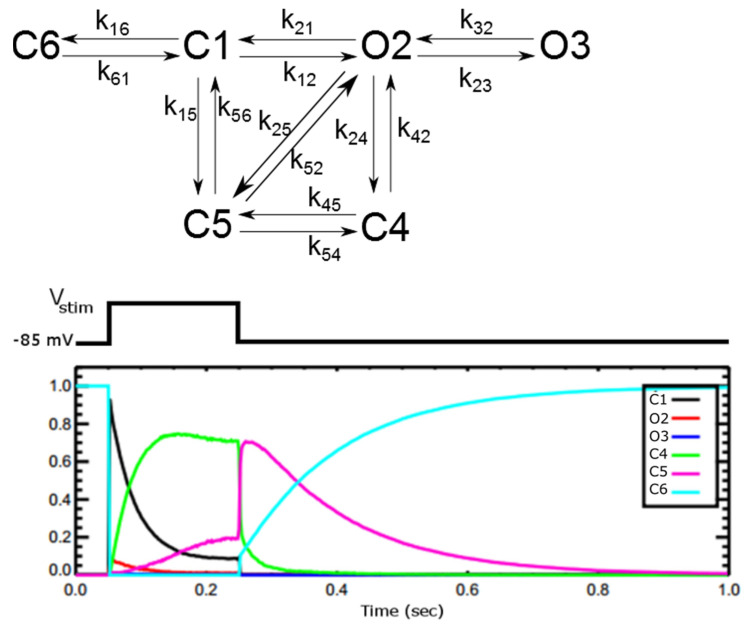
Schematic diagram of the model (**top**) for the L-type calcium channel (DHPR channel) from [29]. C1 and C6 are closed states with C6 the resting state. O2 and O3 are the open states that conduct Ca^2+^ ions with equal conductance. C4 is Ca^2+^-dependent inactivated state. C5 is the voltage-dependent inactivated state. The fraction of LCC channels in different states during the voltage-clamp of V_stim_ = +10 mV for 200 ms (**bottom**).

The 6-state L-type Ca^2+^ channel (LCC) model, Figure 2, is derived from 5-state LCC model for rat ventricle myocyte from Sun and co-workers with parameters adjusted for the new spark model [31,32]. The Sun model was developed to work in the range of −30 mV + 30 mV of the transmembrane potential reproducing single channel dwell times and currents from populations of channels matched to experiments. As Sun and co-workers suggested in their original paper, the 6-th state C6 was added to work with stronger depolarization (<−40 mV), so that all the channels stay in this state when the cell is at rest.

The inactivation of the LCC was modelled via 2 separate pathways: VDI (voltage-dependent inactivation (O2→C5)) and CDI (Ca^2+^-dependent inactivation (O2→C4)). At each release site, the Ca^2+^ level that controls the inactivation is the subspace calcium. The source of contributing calcium to this microdomain is the influx of calcium via LCC and the release of calcium from SR via RyR. The much higher level of calcium in this subspace, i.e., ~100-fold compared to cytosolic bulk calcium, enhances the rate of inactivation of LCC and thereby preventing calcium overload.

The endogenous buffer CaM bound to Ca^2+^ (CaCaM) is the effector for Ca^2+^-dependent inactivation (CDI) of L-type Ca^2+^ channel. The calcium-free CaM is called apo calmodulin (apo-CaM) with two homologous domains, known as lobes [31]. For each apo-CaM molecule, there are four different calcium binding sites: two at the E-F hand motifs of the N-terminal (N-lobe) and two at the E-F hand motifs of the C-terminal (C-lobe) of CaM (for review see [32]). In L-type Ca^2+^ channels (Cav1.2), CDI is triggered by the binding of two Ca^2+^ ions to the C-lobe of CaM [33,34,35]. It means that to model CDI, two Ca^2+^ binding at C-lobe is necessary. This differs from the original Sun model, which used the Hill coefficient as 3 based upon earlier data [36]. In addition, the original model didn’t incorporate the loss of calcium in the subspace due to binding to calmodulin (CaM). This is important as the level of free [Ca^2+^]_ds_ control the gating of RyR2 channels. This was also corrected in our modified model. Experiments suggest the existence of non-junctional DHPR (10–20%), located on the external sarcolemma and not forming the release sites with RyR2 [37,38]. Hence the contribution of calcium from a small fraction of DHPR I_dhpr,nj_ (15%) was also added.

#### 2.1.4. Na^+^ Channel Model

**Figure 3:**
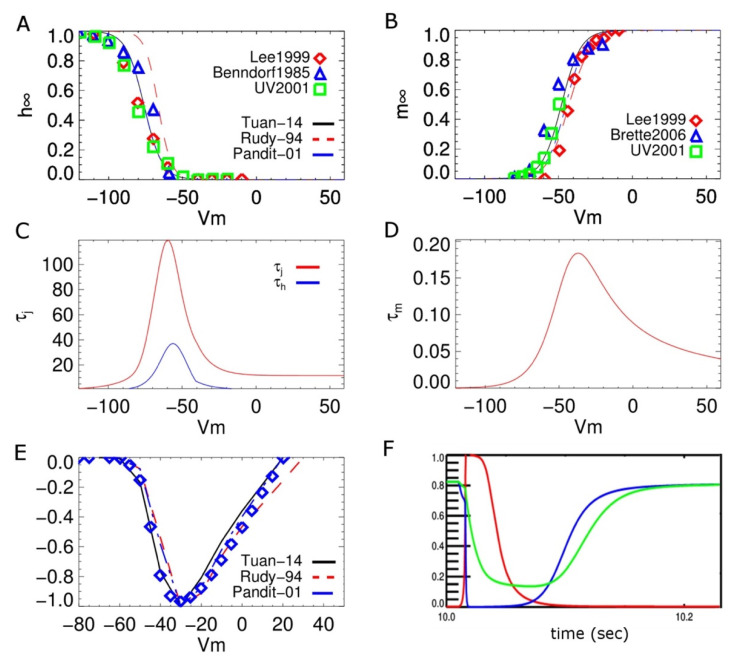
(**A**) steady-state inactivation curve (**B**) steady-state activation curve; (**C**) voltage dependence of slow inactivation gate time constant (**D**) voltage dependence of activation gate time constants at different voltages. (**E**) I-V curve, (**F**) dynamics of gating variables of the proposed model during an AP—activation gate m (red), inactivation gate h (blue), and slow inactivation gate j (green).

The rapid inward Na^+^ current in the cardiac cells exhibits a bi-phasic time courses for recovery during inactivation and one activation gating variable [39]. We have modified the Pandit model so that the model more closely simulates data for the adult rat ventricular myocyte than does the more commonly used Luo-Rudy model as shown in Figure 3 [40,41,42,43,44]. The new model better simulates the correct upstroke velocity for rat as 213.8 ± 6.6 V/s with the overshoot is 48.76 ± 1.09 (mV). Experiments estimate the velocity range as 150–190 V/s at 23 °C V/s in Wistar rat heart which is calculated to be 184–233 V/s using a Q10 of 1.23 [42,45,46]. The new model also better estimates the value of h∞ to be 0.812 at −80 mV at body temperature. This value of h_∞_ has been experimentally estimated as 0.7–0.8 at −80 mV in rat and mice [39,47]. In contrast, the value of h∞ in Pandit is 0.6; and the value in Luo-Rudy is 0.97. Our estimate takes into consideration the temperature dependence for recovery kinetics with the Q10 ranging from 1.7 to 2.3 for membrane potential in the range −76 mV to −62 mV, and 1.5 to 1.8 for the membrane potential in the range −84 mV to −65 mV [43]. The maximum current conductance is 8 mS/cm^2^ which is in the range 3–25 mS/cm^2^ [39,48].

#### 2.1.5. K^+^ Channel Models

There are four different K^+^ channel currents (I_tof_, I, I_K1_, and I_Kss_). The formula for fast and slow transient outward currents (I_Ktof_, I_Ktos_, respectively) are based on the model based on experimental data observed in mice [49].

#### 2.1.6. Sarcoplasmic Reticulum Ion Pumps

The sarco (endo)plasmic reticulum Ca^2+^-ATPase (SERCA) pump re-sequesters Ca^2+^ back to the SR/ER during each excitation-contraction cycle to facilitate muscle relaxation by pumping two calcium ions per ATP molecule hydrolyzed [50]. We used the 2-state formulation by Tran and co-workers developed because it is constrained both by the thermodynamic and kinetic data for the SERCA pump [51].

#### 2.1.7. Calcium Buffers

The three endogenous buffers of calmodulin (CaM), troponin (Trpn), and the phospholipids of the SR membrane (SRbuf) are used for the bulk myoplasm. The troponin complex consists of three different subunits. The troponin complex as modeled includes the binding of calcium (troponin C), the inhibition of actomyosin interaction (troponin I), and the binding to tropomyosin (troponin T).

#### 2.1.8. Membrane Potential

During AP, the dynamics of the membrane voltage *V_m_* are governed by the ionic currents described above.

### 2.2. Numerical Methods

We used our patented Ultra-fast Monte Carlo Simulation Method to solve for the states of the RyR2 and L-type Ca^2+^ channels in each release unit [52]. Using this method the RyR2 channels at each release site are modeled as a single stochastic cluster, and the L-type channels are modeled similarly. Rather than keeping track of individual channel’s state, we used a mean-field approach in which we assume all channels in the cluster see the same local calcium concentration in the dyadic subspace [53,54]. Thus, the individual channel’s states are ignored, and only the number of channels in each state is important. Each release site is fed with a different sequence of pseudo-random numbers. These Monte-Carlo simulations are computed on Fermi-GPU cards, with pseudo-random numbers were derived from the Saru PRNG algorithm implemented on GPU provided by Steve Worley (Private communication at GTC’12) [55]. Instead of using a fixed timestep, an adaptive time-step strategy is used. When the channel fires, a smaller time-step is selected; first to ensure numerical stability, second to limit maximum 10% of the CRUs having state changes to occur at a time [56,57]. This limits Type II error with the hypothesis that there is only channel state transition in the cluster per time step. In fact, when a full Monte Carlo Simulation is performed there are two channels undergoing state transitions in each timestep <0.6% of the time.

The system of ordinary differential equations comprising the model is solved using the explicit Euler method. The small and adaptive timestep (10–100 ns) which is required to simulate the fast and stochastic gating of DHPR and RyR2 channels is sufficient to ensure numerical stability.

## 3. Results

**Figure A1:**
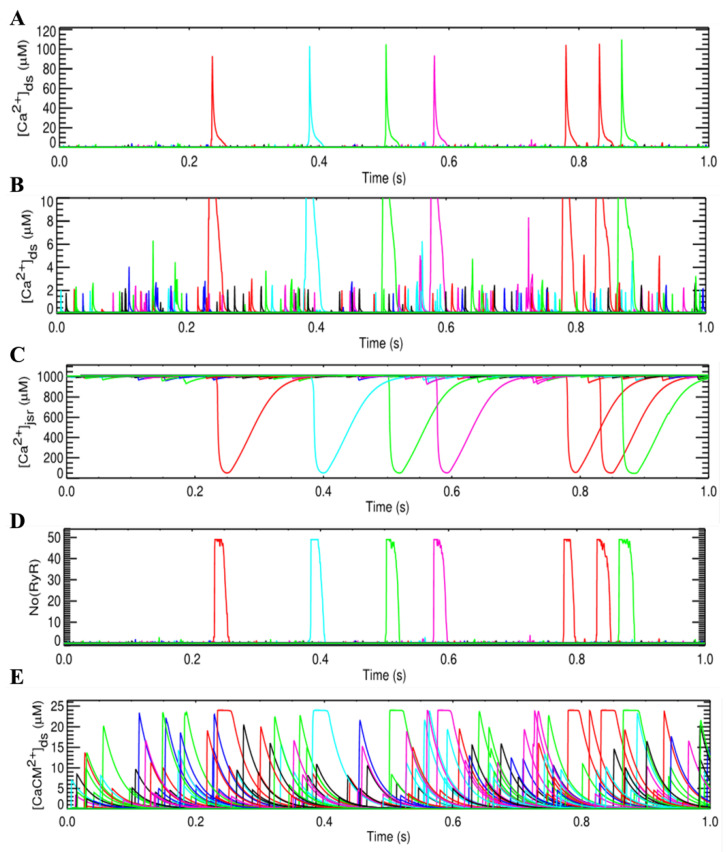
Calcium in the subspaces during (**A**) Ca^2+^ sparks and (**B**) Ca^2+^ quarks, (**C**) Dynamics of jSR Ca^2+^ release, (**D**) Number of RyR openings, (**E**) Ca^2+^-bound calmodulin complex.

**Figure A2:**
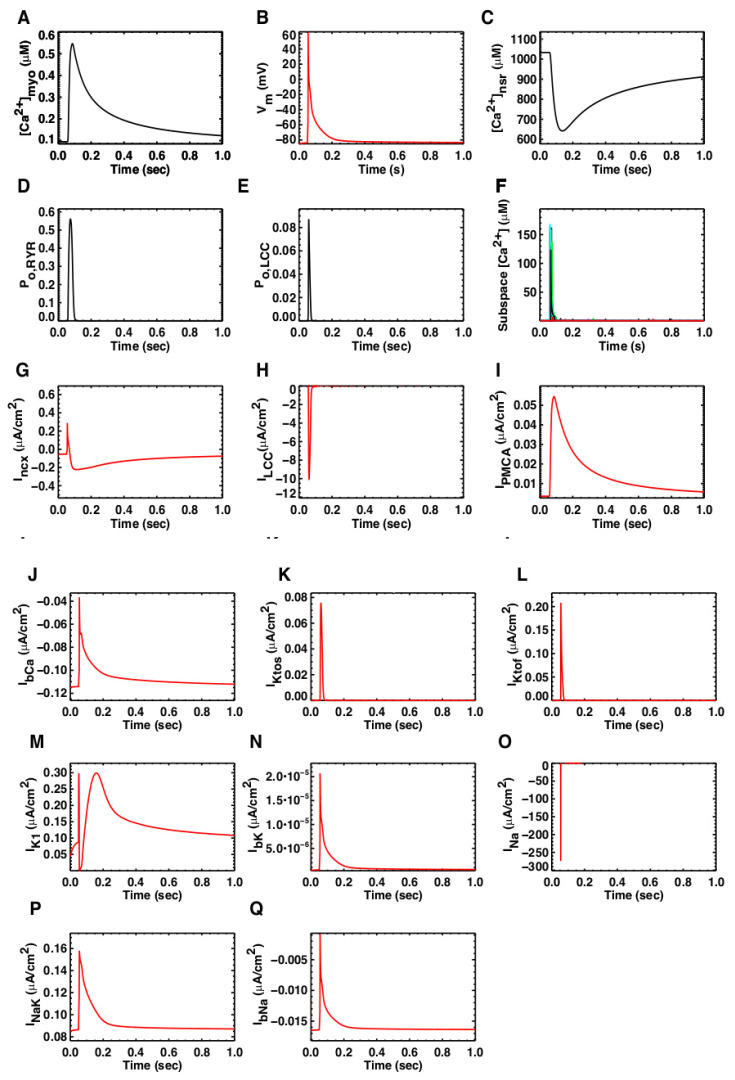
A single action potential at 1 Hz pacing. To emulate the excitation-contraction of the cell, a stimulus current Istim = −6.5189 µA/cm^2^ was given during 5.0 ms second [40]. (**A**) [Ca^2+^]_myo_, (**B**) Membrane potential (V_m_); (**C**) Network SR Ca^2+^ concentration ([Ca]_nsr_); (**D**) RyR2 open probability (P_O_,RyR); (**E**) L-type Ca^2+^ channel open probability (P_O_,LCC); (**F**) subspace free Ca^2+^ concentration ([Ca^2+^]_ds_); (**G**) L-type Ca^2+^ current (I_LCC_); (**H**) NCX current (I_ncx_); (**I**) PMCA current (I_PMCA_); (**J**) Ca^2+^ current (I_Na_); (**K**) Na/K exchanger current; (**L**) K_1_ current; (**M**) slow component of transient-outward K^+^ current; (**N**) fast-component of transient-outward K^+^ current; (**O**) Na^+^ current; (**P**) Na^+^-K^+^ ATPase current; (**Q**) Background Na^+^ current.

The model integrates the complex mechanisms involved in excitation-contraction coupling by describing the 20,000 stochastic calcium release units. In the model components were validated in the model described above and the model dynamics below in the results section. For example, the model demonstrates the same mechanism of release as our previous work and fully accounts for the SR Ca^2+^ visible and invisible leak by flux through the RyR2 channels in the forms of Ca^2+^ sparks and non-spark openings, respectively (Figure A1) [27,58,59]. Details of the ionic currents are shown in Figure A2.

### 3.1. Dynamics of Calcium during a Twitch-Relaxation Cycle

**Figure 4:**
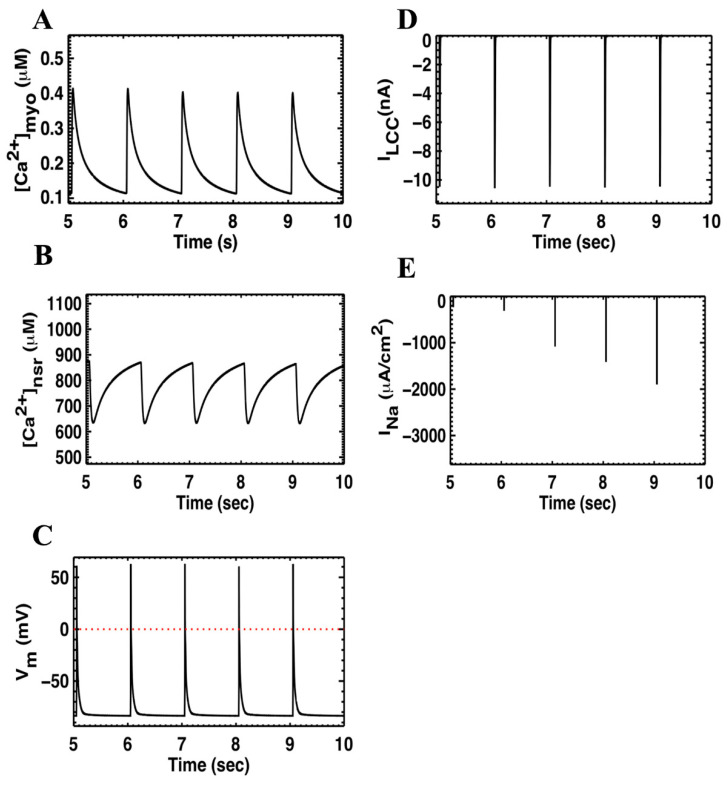
A long trace of calcium dynamics in the (**A**) myoplasmic [Ca^2+^], (**B**) network SR [Ca^2+^], and (**C**) Action Potential, (**D**) L-type current density, and (**E**) Sodium current density.

Figure 4 shows for 1 Hz pacing the time courses for a train of action potentials, myoplasmic calcium transients, network, and SR calcium transients. In our model, the ratio of SR calcium release over the influx of calcium during a twitch is 10.0 ± 0.3. It means that, on average, the SR-release contributes about 90.07% and calcium influx contributes 9.03%. This approximates the value 92% of SR contribution estimated for rat ventricular myocytes [9,60]. During one cycle, our model produced a total extrusion of calcium of about 11.5 µM/cell/s. Among this, ~1 µM is extruded by the PMCA and ~10.5 µM is extruded by the NCX. This is in agreement with the value given above by [61]. The ionic currents during a twitch-relaxation cycle are given in Figure A2. The model also simulates pacing, with APD20 ~10.69 ms, APD50 ~22.57 ms and APD90 ~78.3 ms as is evident in Figure 4.

### 3.2. Mechanisms of Alternans

**Figure 5:**
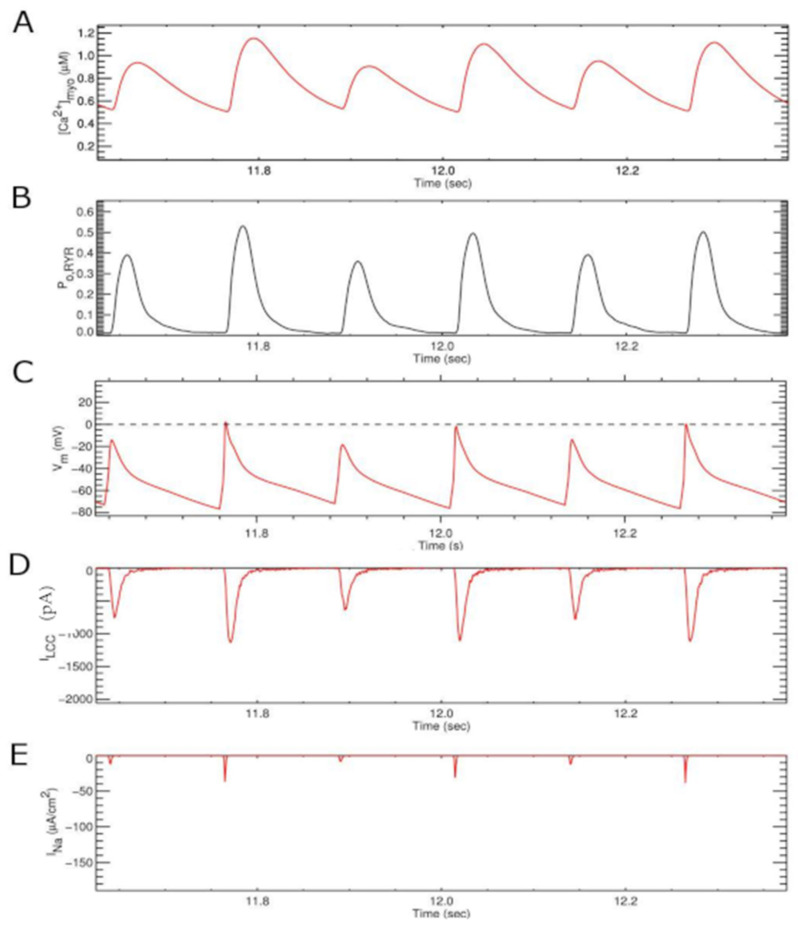
Calcium alternans at 8 Hz: (**A**) Cytosolic calcium, (**B**) RyR open probability Po (RyR2), (**C**) membrane potential V_m_, (**D**) L-type Ca^2+^ current I_CaL_, (**E**) Sodium current I_Na_.

The model produces stable action potential and calcium transient trains below 8 Hz, however, above 8 Hz pacing frequency, periods of alternans are observed. One such period of AC concordant alternans is shown in Figure 5 with both [Ca^2+^]_myo_ and V_m_ alternating in phase. There are also alternating small and large amplitude and duration of other observable quantities such as [Ca^2+^]_SR_, RyR P_O_, and I_Ca_. It is also important to note that the amplitude of I_Na_ and I_CaL_ are greatly reduced.

Note that both the [Ca^2+^]_myo_ and [Ca^2+^]_SR_ fail to recover back to the normal diastolic level between systoles.

Many studies have suggested that alternans is a defect of the calcium subsystem. The conceptual framework of refractoriness, randomness, and recruitment has been used to understand alternans [62]. To study the refractoriness of calcium release beat-to-beat changes of the CRU’s states were analyzed. A CRU is activated if a Ca^2+^ spark occurs. The CRUs are grouped into either activated or inactivated at each beat. Therefore, when considering a pair of beats, there are 4 groups: act-act, act-inact, inact-act, and inact-inact.

**Figure 6:**
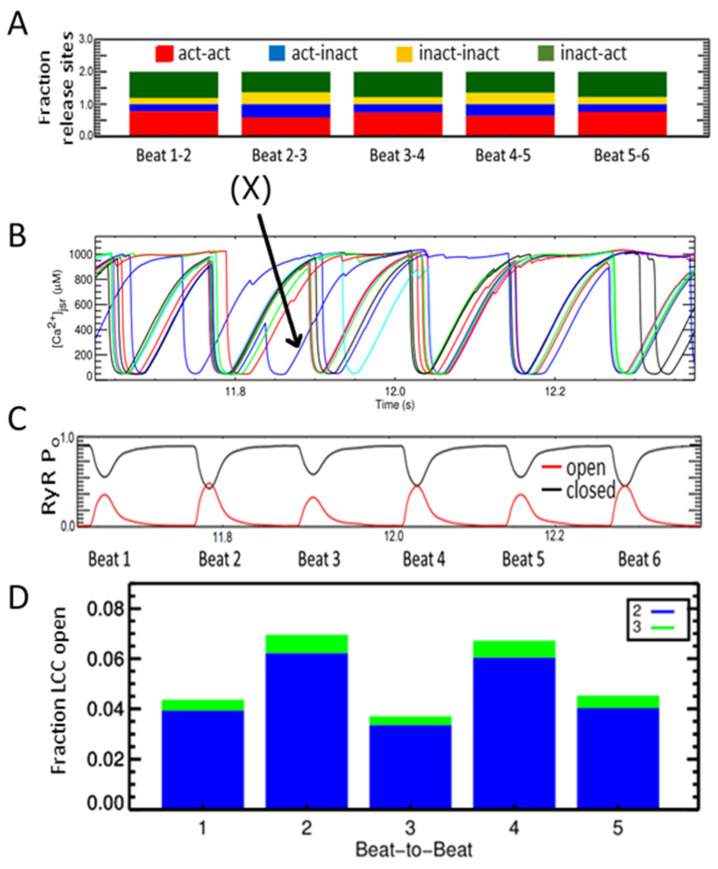
Alternans occurs at 8 Hz pacing rate. (**A**) An example that show a CRU that fires at two contiguous beats, e.g., (X) marks an individual release site that is Act-Act. (**B**) Alternans in the calcium release seen by the changing amplitude and duration of the [Ca^2+^]_myo_. Beat-to-beat variation in CRU’s states where we examine act-act—the fraction of CRU that activate in beat (*i*) (red) and continue to activate in beat (*i* + 1), similarly with act-inact (blue), inact-inact (yellow), and inact-act (dark green). (**C**) The probability of RyR opening at each beat (red = open, black = closed). (**D**) The fraction of LCC open (states O2-blue and O3-green) during each beat.

In the transition from beat-1 to beat-2, there is a large fraction of CRUs changing from Inactivated to Activated (green), and also a large fraction of CRUs which are Activated in beat-1 continue to Activate in beat-2 (red)–the beat that has a strong contraction. For example, the number of activated release sites increases from Beat1-2 as indicated by the increased green and red. Corresponding to this, Figure 6B shows that beat 2 has a higher RyR open probability than beat-1. This suggests that alternans might originate at the level of the CRU. Figure 6C tracks the [Ca^2+^]_jsr_ local depletion for individual CRUs as shows that the CRUs (red, blue, green, and black) activate at alternating beats.

**Figure 7:**
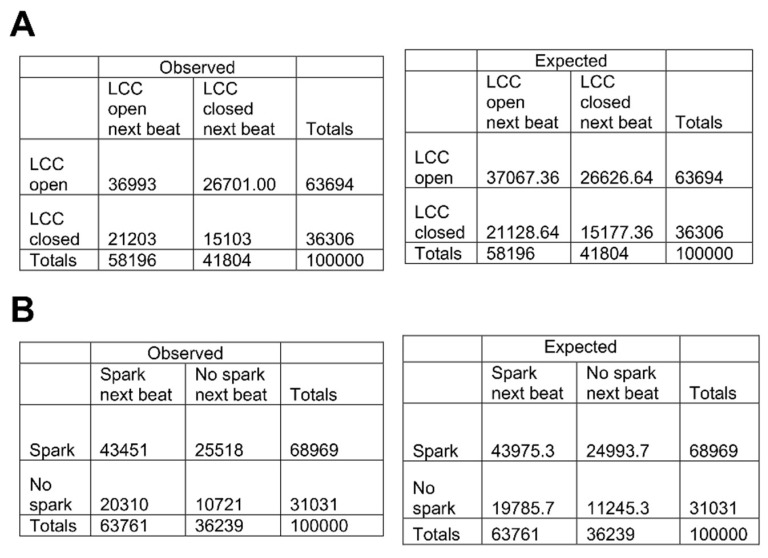
Contingency Tables for Spark Analysis. (**A**) Does L-type Ca^2+^ channels (LCC) opening effect LCC opening in the subsequent beat? (**B**) Does a Ca^2+^ spark in one beat affect Ca^2+^ spark occurrence in the subsequent beat?

However, a Chi-squared test with the 2 × 2 contingency table shown in Figure 7A did not support this hypothesis (*p* < 0.61). Even though there are more L-type Ca^2+^ channels assuming the Ca^2+^ dependent inactivated state during 8 Hz pacing, their number does not exceed 2 or 3 per CRU out of the 7 available channels. This leaves sufficient number of channels in a state to be activated during the subsequent beat. The next hypothesis is that after a CRU has been triggered it is less likely to activate again in the following beat. To quantify this hypothesis, whether or not a spark occurred in the first beat versus whether or not a spark occurred in the second beat, we used a Chi-squared test with a 2 × 2 contingency table (Figure 7B). With a Chi-squared statistic of 55.6 and a *p*-value of *p* < 0.001, this showed that if a spark occurred in the first beat for a given CRU, there was a slightly lower probability that it would occur in the next. Figure 6B depicts the explanation for this. The jSR depletes after a CRU triggers and does not fully recover until the next beat. This lowers the likelihood of CRU activation both through the effects of jSR calcium on RyR open probability and in the event if a RyR does open, the reduced flux will be insufficient to trigger adjacent RyRs. As a result, the model suggests that refractoriness in jSR is an important component of alternans.

**Figure 8:**
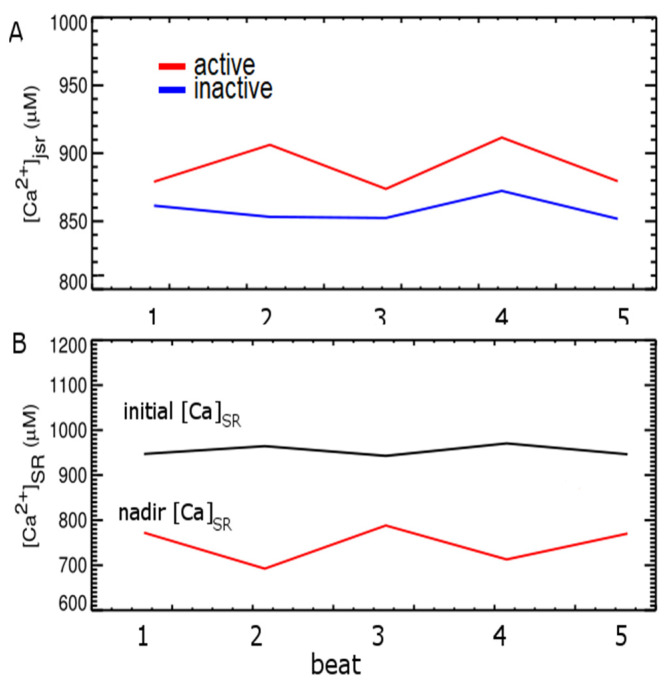
(**A**) The diastolic [Ca^2+^]_jSR_ right before the stimulus from CRUs in two groups: red = Act, blue = Inactivate. (**B**) The upper black line shows the average [Ca^2+^]SR level right before the Istim is applied, and the lower red line shows the nadir of average [Ca^2+^]SR level at the corresponding beat.

These results suggest that the recovery of junctional SR Ca^2+^ after a release event can contribute to the development of alternans. To assess the SR Ca^2+^ content, we split the CRUs at each beat in two groups: those that are activated (Act) and those are not activated (Inact) and investigated two things: (1) the values of [Ca^2+^]_jSR_ at these release sites, right at before the next beat to start, (2) the nadir of [Ca^2+^]_SR_ during calcium release (Figure 8). Larger SR [Ca^2+^] comes with larger Ca^2+^ release and the beats with larger release have more activated CRUs. We can see that the ensemble initial [Ca^2+^]_SR_ is not an indicator of alternans formation. If we look at the nadir [Ca^2+^]_SR_, the result shows a similar small beat-to-beat alternation, in agreement with the study of [16].

**Figure 9:**
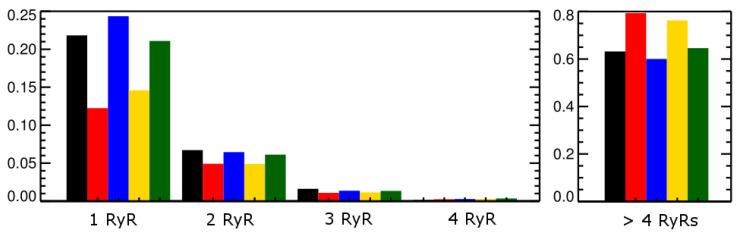
The fraction of CRUs that has 1 or more RyR openings during each beat (black = beat-1, red = beat-2, blue = beat-3, yellow = beat-4, green = beat-5).

The mechanism by which randomness of calcium release arises in the system is also explained by the model. The reduction in I_Na_ current which reduced the action potential magnitude contributed a huge effect in reducing the number of L-type channels opening that opens the gateway for alternans (Figure 5). When there is less I_Na_ (Figure 5E), there is a smaller AP (Figure 5C) and reduced L-type current (Figure 5D). These beat also have a smaller RyR open probability (Figure 5B) and Ca^2+^ transient (Figure 5A) This is in agreement with the result in which a small depolarizing potential under voltage clamp can induce alternans [16], and confirms the hypothesis that SR Ca^2+^ release is graded with the beat-to-beat alternation in I_CaL_ [63,64]. The reduction in L-type opening reduces the synchronization of activation of the CRUs. Furthermore, the elevated myoplasmic [Ca^2+^] increases the stochastic activation of Ca^2+^ sparks. At the normal quiescent condition, as shown in our previous study, the activation of a CRU is the result of 8–10 RyR2 opening [27]. During field-stimulus simulation with high pacing, this number reduces to 5, where Ca^2+^ alternans is observed (Figure 9). During the large beat, there can be up to 80% of CRUs activated, though the time to peak of activation is slower, e.g., 60%.

**Figure 10:**
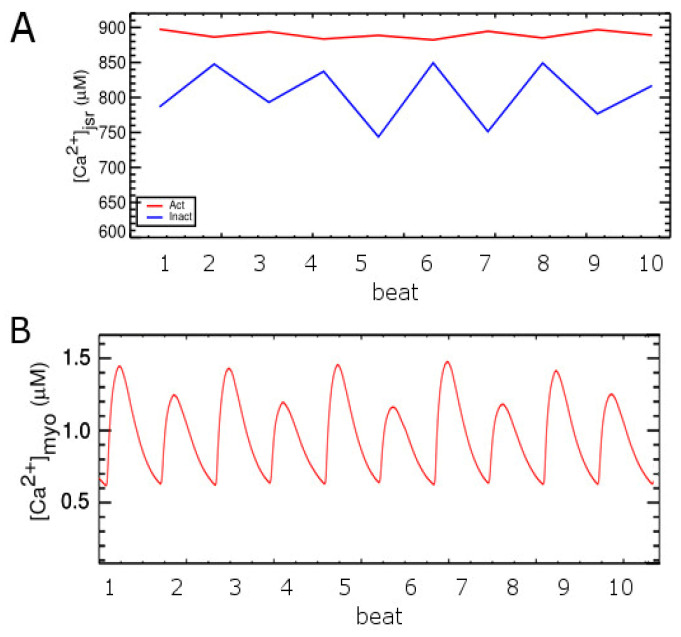
8 Hz pacing with [Na^+^]_i_ = 15 mM: (**A**) The average of [Ca^2+^]_jSR_ right before the stimulus from CRUs in two groups: red = Act, blue = Inactivate, and the nadir value. (**B**) Alternans in the myoplasmic calcium transient.

We then explored the hypotheses that a high level of [Na^+^]_i_ increases the likelihood of alternans through the modulation of Na^+^/Ca^2+^ exchanger activity (NCX). The normal function of NCX is to extrude one calcium ion in exchange for three sodium ions. It has been hypothesized that at high level of [Na^+^]_i_, it may fail to extrude calcium, thus maintaining a high diastolic cytosolic [Ca^2+^]. In other words, increasing Na^+^ lead to more reverse mode NCX, leading to increases [Ca^2+^]_myo_ and [Ca^2+^]_SR_. With 8 Hz pacing simulation with our model, alternans were observed at [Na^+^]_i_ at ~10.2 mM. We tested the effect of high sodium concentration by fixing the sodium level with [Na^+^]_i_ = 15 mM. Under this condition, alternans were also observed yet the levels of cytosolic calcium, at both basal and peak, are higher (Figure 10).

**Figure 11:**
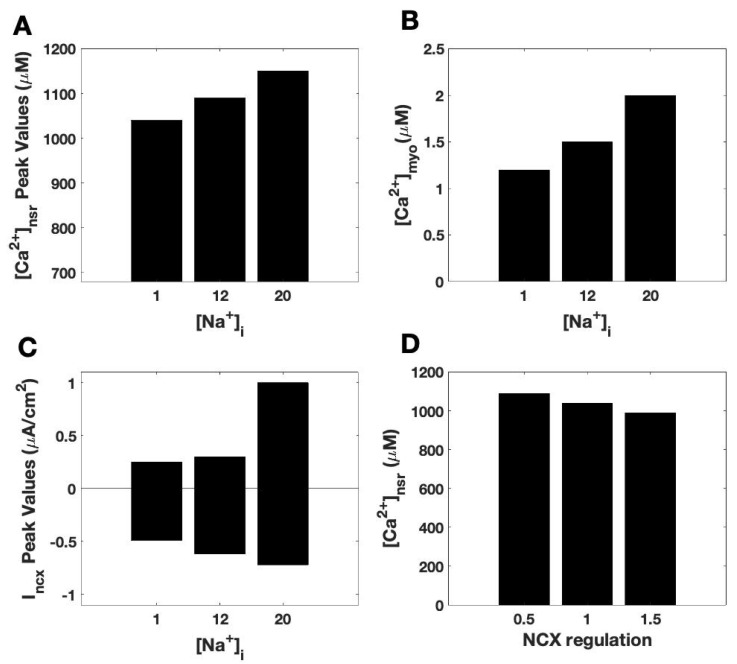
Tables for analyzing the dependence of Calcium dynamics on [Na^+^]_i_ and Na^+^/Ca^2+^ exchanger activity (NCX). (**A**) Peak network SR Ca2 concentration ([Ca^2+^]_nsr_) at different myoplasmic Na^+^ concentrations ([Na^+^]_i_). (**B**) Peak myoplasmic Ca^2+^ concentration ([Ca^2+^]_myo_) at different values of [Na^+^]_i_ and (**C**) Peak forward and reverse Na^+^/Ca^2+^ exchanger (NCX) current at different values of [Na^+^]_i_. (**D**) Peak [Ca^2+^]_NSR_ at different Na^+^/Ca^2+^ exchanger (NCX) activity levels.

Simulations were performed with [Na^+^]_i_ held at 1 mM, 12 mM, and 20 mM. As [Na^+^]_i_ increase the diastolic (peak) [Ca^2+^]_nsr_ and the [Ca^2+^]_myo_ both increase (Figure 11A,B). As expected, with increasing [Na^+^]_i_ the reverse NCX current increases bringing more Ca^2+^ into the cell (Figure 11C). The forward NCX (Ca^2+^ extrusion) also increases due to the higher [Ca^2+^]_myo_. When the NCX rate is increased intracellular [Ca^2+^] falls due to the increase extrusion (Figure 11D). Increasing [Na^+^]_i_ also attenuates alternans (Figure A4 and Figure A5).

**Figure 12:**
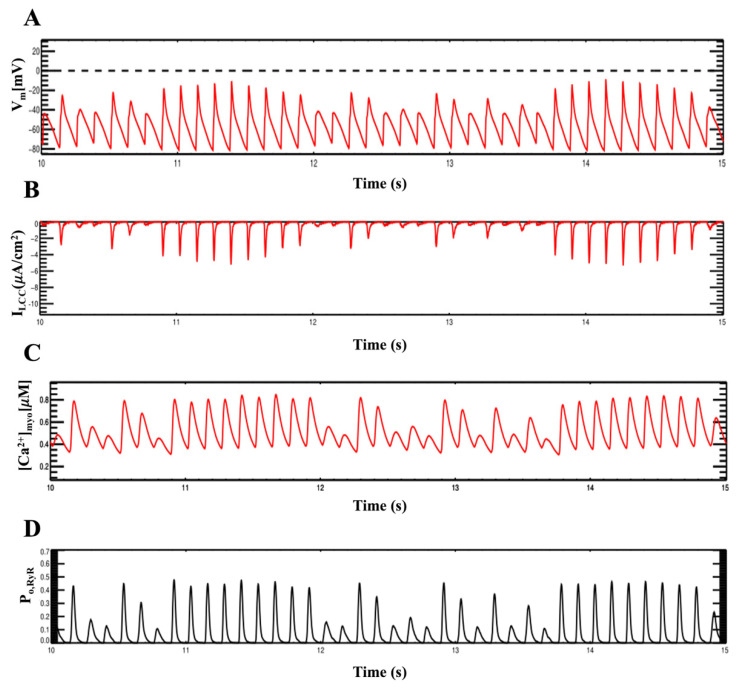
Alternans at high pacing rate (8Hz) using a stochastic Na^+^ current model at 23 °C, (**A**) action potential. (**B**) I_CaL_ current. (**C**) cytosolic calcium. (**D**) opening probability of whole-cell ryanodine receptors.

If reduction of NCX activity by high Na^+^ is crucial for the formation of alternans, NCX upregulation should reduce alternans. Consistent with this hypothesis, high expression levels of NCX reduces SR calcium, along with cytosolic [Ca^2+^] and thus reduce alternans amplitude (Figure 12C). Large reduction of NCX (to 50% control) still generates alternans, however, the calcium alternations are attenuated. It again confirms that there is a range of changes that can be critical for the arrhythmogenesis to occur. When the temperature is reduced (23 °C), the alternans become more prominent (Figure 12). In this test case, the simulation was done using a stochastic model for the Na^+^ current developed by [40]. This is in agreement with many other experiments that low-temperature increases the likelihood of alternans development [65,66,67], and we anticipated that Na^+^ current plays an important role here.

## 4. Discussion

Cellular alternans in cardiac myocytes have been shown in experiments and modeling to have a mechanism that depends both on the membrane currents and on the Ca^2+^ subsystem. This modeling study demonstrates that for alternans produced at high pacing rates, both mechanism act synergistically to produce alternans. Under the hypothesis that relies on membrane currents, action potential restitution is the underlying cause of cardiac alternans [9,10,11,12,13]. Studies that have suggested that modified intracellular calcium cycling plays a role in occurrence of mechanical and electrical alternans have found that it is possible to have alternans in calcium release without requiring action potential alternans [14,15,16,17,18,19,20,21]. Current computational models are unable to recreate this phenomenon; unless certain modifications to the ionic currents were made [9,23]. Newer studies have suggested that both mechanisms are possible under different conditions [24,25].

Computational studies have suggested that the randomness of Ca^2+^ sparks; recruitment of a Ca^2+^ spark by neighboring Ca^2+^ sparks; and refractoriness of Ca^2+^ release units are the important factors required for cardiac alternans [68]. The work here is consistent with this idea. While we take into account the stochastic nature of ionic channel gating at individual release site, the subspace area is treated as a single compartment and thus all ion channels (RyRs and LCCs) in the same calcium release site sense the same level of calcium. The potential spatial distribution of the receptors that may govern the allosteric interaction between them, underlying the long- or short-range correlation mechanism between receptors is approximated using a mean-field approach which governs the non-linearity via a non-linear coupling function as shown in Appendix B.2. One question that can be probed using the model is how Ca^2+^-dependent inactivation of the L-type Ca^2+^ channel varies beat to beat during alternans. Figure 6D shows more L-type Ca^2+^ during large beats than small beats during alternans. If this is caused by increased Ca^2+^ dependent inactivation, it would be expected that there be a faster rate of decay of the L-type current during the large currents. The rate of L-type current decay is shown in the Figure A6 in Appendix A by overlapping the currents at a big and small beat during alternans. Fitting an monoexponential curve (f(x) = a × exp(−x/b)) to the decay yielded a = −9.37 pA and b = 0.21 s for the large beat and a = −55.8 pA and b = 0.46 s for the small beat with a R2 = 0.977 and R2 = 0.980, respectively. The large beat where Ca^2+^ is more elevated displays a faster decay than the small beat.

Refractoriness in Ca^2+^ release is demonstrated to be crucial to alternans by our statistical analysis of sparks at each release site (Ca^2+^ release unit). Our studies also show that an uncoupling of depolarization to Ca^2+^ release occurs as the L-type current is reduced both through Ca^2+^-dependent inactivation and smaller action potential amplitude due to reduced Na^+^ current. Our model is not a spatial model and hence does not model neighboring sparks. However, we do see recruitment as elevated bulk myoplasmic Ca^2+^ during rapid pacing, in part produced by more sparks, promotes Ca^2+^ sparks at additional Ca^2+^ release units.

**Figure A3:**
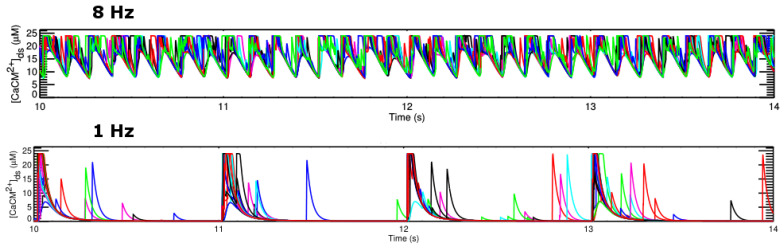
The amount of Ca^2+^/CaM complex in the subspace is greater during pacing at 8 Hz compared to acing at 1 Hz pacing leading to increased Ca-dependent inactivation of the L-type channel.

**Figure A4:**
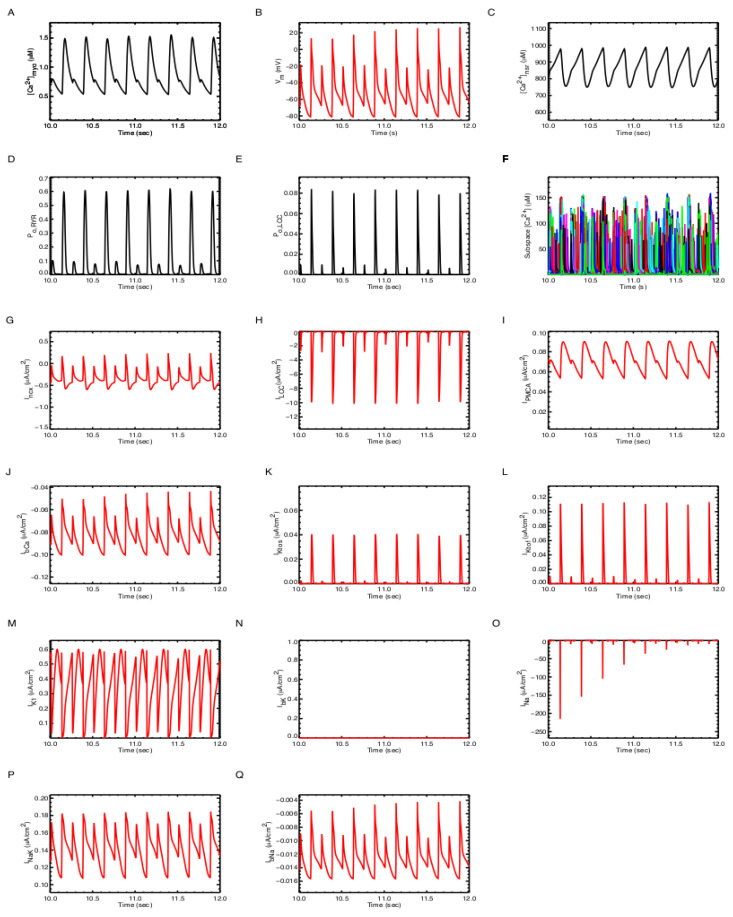
Pacing at 8 Hz pacing produces alternans and a diastolic [Na^+^]_i_ of 10.2 mM. To emulate the excitation-contraction of the cell, a stimulus current I_stim_ = −6.5189 µA/cm^2^ was given during 5.0 ms second [40]. (**A**) [Ca^2+^]_myo_; (**B**) Membrane potential (V_m_); (**C**) Network SR Ca^2+^ concentration ([Ca^2+^]_nsr_); (**D**) RyR2 open probability (P_O_,RyR); (**E**) L-type Ca^2+^ channel open probability (P_O_,LCC); (**F**) subspace free Ca^2+^ concentration ([Ca^2+^]_ds_); (**G**) L-type Ca^2+^ current (I_LCC_); (**H**) NCX current (I_ncx_); (**I**) Plasmalemmal Ca^2+^-ATPase current (I_PMCA_); (**J**) background Ca^2+^ current (I_Cab_); (**K**) slow transient outward current (I_tos_); (**L**) fast transient outward current (I_tof_); (**M**) K_1_ current (**M**) slow component of transient-outward K^+^ current; (**N**) Background K^+^ current; (**O**) Na^+^ current; (**P**) Na^+^-K^+^ ATPase current; (**Q**) Background Na^+^ current.

**Figure A5:**
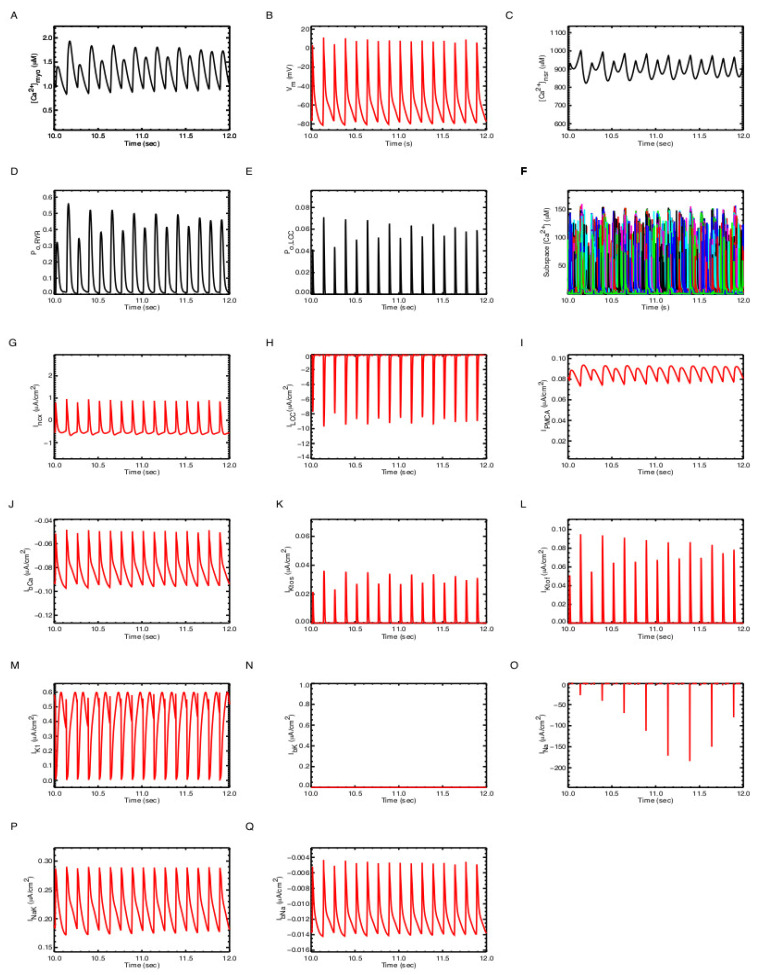
Pacing at 8 Hz pacing produces alternans with diastolic [Na^+^]I = 20 mM. To emulate the excitation-contraction of the cell, a stimulus current I_stim_ = −6.5189 µA/cm^2^ was given during 5.0 ms second [40]. (**A**) [Ca^2+^]_myo_; (**B**) Membrane potential (V_m_); (**C**) Network SR Ca^2+^ concentration ([Ca^2+^]_nsr_); (**D**) RyR2 open probability (P_O_,RyR); (**E**) L-type Ca^2+^ channel open probability (P_O_,LCC); (**F**)subspace free Ca^2+^ concentration ([Ca^2+^]_ds_); (**G**) L-type Ca^2+^ current (I_LCC_); (**H**) NCX current (I_ncx_); (**I**) PMCA current (I_PMCA_); (**J**) Ca^2+^ current (I_Na_); (**K**) Na/K exchanger current; (**L**) K1 current; (**M**) slow component of transient-outward K^+^ current; (**N**) fast-component of transient-outward K^+^ current; (**O**) Na^+^ current; (**P**) Na^+^-K^+^ ATPase current; (**Q**) Background Na^+^ current.

**Figure A6:**
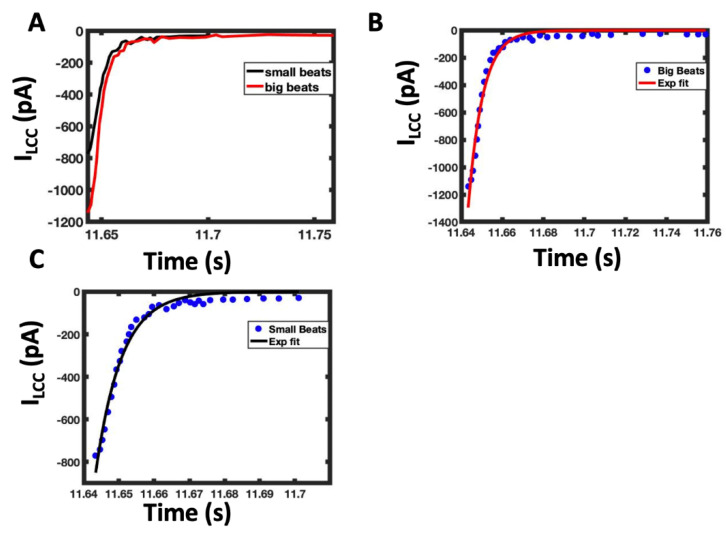
Ca^2+^-dependent inactivation of the L-type channel occurs at different rates in large and small beats during alternans. (**A**) The decay in the L-type Ca^2+^ current for large (red) and small (blue) beats. (**B**) Fitting an monoexponential curve (f(x) = a × exp(−x/b)) to the decay yielded a = −9.37 pA and b = 0.21 s for the large beat with a R2 = 0.977. (**C**) Fitting an monoexponential curve (f(x) = a × exp(−x/b)) to the decay yielded a = −55.8 pA and b = 0.46 s for the small beat with a R2 = 0.980, respectively.

Previous studies have shown that block of NCX by ORM-10962 has been shown to attenuate alternans in experiments [69]. In our model with decreasing NCX activity the NSR Ca^2+^ load increases and NCX attenuates alternans. The model suggests that large reduction of NCX (to 50% control) still generates alternans, however, the calcium alternations are attenuated. This suggests that there is a range of conditions that can be critical for the occurrence of alternans. Other studies have shown that blocking late Na^+^ current by ranolazine attenuates alternans, presumably by reduction of reverse NCX although this has not been proven [70,71]. While reduction of NCX, can reduce Ca^2+^ entry through reverse-NCX, it will suppress the greater role of Ca^2+^ extrusion leading to more calcium overload. The rat ventricular myocyte does not have a late component of the Na^+^ current so we cannot test this directly. In our simulations, increasing intracellular Na^+^ to 12, 15, and 20 mM, the NSR Ca^2+^ load increases, yet alternans are attenuated. There is a decrease in the action potential duration with increasing [Na^+^]_i_ as shown in the Figure A3, Figure A4 and Figure A5 allowing for recovery of the Na^+^ current and production of a regularization of the action potential. It suggests that blocking late Na^+^ current allows the Na^+^ channels to recover to open in the next beat resulting in attenuated alternans.

Calcium sensitive K^+^ channels (SK channels) have been found in rat ventricular myocytes. These channels activate and allow hyperpolarizing outward current when [Ca^2+^]_myo_ is elevated with a K_0.5_ = 0.5 µM [72]. The SK channel conductance in rat peaks at 1–2 pA/pF [73]. This Ca^2+^ sensitivity is conferred though the binding of Ca^2+^ with calmodulin with experimental studies suggesting calmodulin variants can alter SK channel function and potentially lead to arrhythmia [74]. Furthermore, SK channel expression has been observed to increase during heart failure or after myocardial infarction and have been suggested to contribute to arrhythmia [75,76]. Computational rabbit ventricular myocyte models have suggested that when these channels are blocked, a mild to moderate action potential prolongation occurs depending on the conductance level assigned to the channel [73,76]. However, at the physiological conductance levels, these models predict a very small effect. Additionally, experimental studies have und AP clamp suggested that blocking the K^+^ using Cs under AP clamp, resulted in no change in the difference current between beats in alternans suggesting little role for the SK current alternans. The same study however found that the Ca^2+^-activated Cl^−^ current did play a role [77]. For this reason, we did not include them in the current model and left them for future studies of arrhythmia and disease.

Calmodulin has high and low affinity Ca^2+^ binding sites. Experiments have measured high affinity sites in the C-lobe to have a K_d_ = 1 µM and the low affinity sites in the N-lobe to have a K_d_ = 12 µM [78,79]. We have chosen to only include the low affinity N-lobe site because in the microdomain near the L-type channel upon L-type opening that high affinity site will saturate rapidly leaving the low affinity site to play the regulatory role. This is supported by the role the N-lobe has been found to play in Ca^2+^ dependent inactivation of the L-type Ca^2+^ channel [80]. Furthermore, at high pacing rates where Ca^2+^ is elevated, it even more likely that the C-lobe is saturated and plays less of a regulatory role. We did not choose to use the model by Limpitikul et al., which uses a C-lobe K_d_ = 1.15 µM and a N-lobe K_d_ = 0.9 µM as it would not be appropriate for a model which explicitly models the dyadic subspace [81]. According to Uniprot, the D96V, D130G and F142L variants are implicated in LQTS. While located in the C-terminus, molecular dynamics simulations show that they affect the positional relation between the lobes such as the linker distance and dihedral angles between the lobes, so the effect of the variants is not simply an effect on Ca^2+^ binding affinity [82]. Including the low affinity sites would not affect the model results presented here. However, to model LQTS all four sites would need to be considered. This is left for future work.

The model is a set non-linear differential equations with stochastic elements. The conversion to alternans is period doubling behavior and has been observed both in experiments and in other models of the cardiac action potential [83,84,85]. For example, with rapid pacing the dog heart can develop alternans. With increasing pacing rate there is period doubling, a repeating sequence of four beat amplitudes. With further increases, fibrillation or chaos occurs. We have also observed this behavior in our previous Guinea pig model and this model (at 12 Hz) [86].

Ca^2+^ oscillations have been observed in cardiac ventricular myocytes under Ca^2+^ overload conditions. Similar to deterministic systems such as our previous model, this model is capable of Ca^2+^ oscillations due to the dynamics of the Ca^2+^ subsystem because it is a non-linear excitable system [86]. However, the cardiac ventricular myocyte is a driven system with a periodic applied current to trigger action potentials similar to experiment that mimic the periodic excitation of a ventricular myocytes by adjacent cells during the heartbeat. If the rapid pacing is abruptly ceases, there will be some spontaneous Ca^2+^ release events (calcium oscillations) similar to experiment [87]. Furthermore, we have developed a spatial model of the rat ventricular myocyte that displays oscillatory Ca^2+^ waves under calcium overload conditions with no depolarization stimulus similar to the phenomena observed in isolated rat cardiac ventricular myocytes [88,89].

Anomalous diffusion has been proposed to explain the spatial diffusion of Ca^2+^. Anomalous diffusion by definition is a diffusion where the mean squared displacement does not depend linearly on time [90]. This is different from the Fickian diffusion that causes Brownian motion which has been observed in cells. Other modeling studies have invoked anomalous diffusion to create Ca^2+^ spark models because existing models using Fickian diffusion have only achieved FWHM of 1.0 µm compared to the experimentally observed FWHM of 1.8 µm [91]. With their anomalous diffusion model (subdiffusion) it is possible to get a FWHM of 2.0 µm. However, we have shown previously that with our model that includes Fickian diffusion we can get a FWHM of 1.8 µm [89]. In our modeling efforts there has been no need to invoke novel diffusion formulations to reproduce experimentally observed phenomena.

## 5. Conclusions

In conclusion, we presented here a study using a stochastic computational model to study the cellular mechanism underlying cardiac alternans in rat ventricular myocyte. The model helps to explain a modest role of [Ca^2+^]_jSR_ in forming alternans, while it’s suggested that disturbing I_Na_, I_CaL_ and membrane potential plays a dominant role in the forming of pulsus alternans. In addition to this, the model was able to reproduce results at conditions that have been known for alternans like lowering the temperature, high [Na^+^]_i_ or reducing alternans amplitude by up/down regulation of NCX. The limitation of the model is the inability to investigate the spatial effect on the generation of cardiac alternans, i.e., subcellular Ca^2+^ alternans or calcium waves in alternans. This will be the next step in our study with a full-scale spatio-temporal model of the cardiac ventricular myocyte to investigate subcellular Ca^2+^ alternans [92,93,94].

## Data Availability

Model codes are publicly available at the Mason Archival Repository Service (MARS) at the following link: available online: https://hdl.handle.net/1920/11957 (accessed on 5 October 2021).

## Appendix A

### Appendix A.1. Spark Termination

The termination of Ca^2+^ sparks involves the combination of luminal calcium depletion, which reduce the driving force for the Ca^2+^ flux; the stochastic closing of the channels and their failure to re-open due to less subspace and jSR Ca^2+^, and the effect of the increasing number of closed channels on the open ones through allosteric coupling, as in our previous model (Figure 8) [27,58]. Clearly, this predates the concept of pernicious attrition and induction decay in which the smaller Ca^2+^ flux at each RyR2 due to local luminal calcium depletion devastates inter-RyR2 CICR [95]. In fact, the idea the reduced Ca^2+^ flux through the RyR2s in the dyad are insufficient to sustain CICR as suggested in pernicious attrition has been suggested previously [53,86].

### Appendix A.2. Dynamics of Calcium Sparks and Calcium Leak

The model predicts three possible mechanisms of the SR Ca^2+^ leak that are sufficient to account for balancing Ca^2+^ uptake via SERCA2a during the diastolic phase, similar to our previous work [27]. They are visible Ca^2+^ sparks (~120 sparks/cell/s under quiescent), invisible Ca^2+^ leaks known as Ca^2+^ quarks (~4300 quarks/cell/s under quiescent) and calcium leak via a small population of RyRs at a distance from the release sites, known as non-junctional RyR or ‘rogue’ RyR (Figure 8) [96]. The so-called non-spark events or invisible Ca^2+^ quarks are the result of one or a few RyR channels opening whose signals cannot be detected using standard confocal imaging microscopies [97].

The visible leak i.e., 120 Ca^2+^ sparks per cell per second is in the physiological range (100–200 sparks/cell/s) and accounts for 75% of the leak. Part of the invisible leak results from the stochastic gating of one or a few RyR2 channels, accounting for 25% of the leak. This yields a spark fidelity of ~2.8% which is the ability a single calcium release to trigger the Ca^2+^ spark. The total quiescent leak rate was ~1.2 µmol/(L cyt.)/s which is in the range 0.5–5 µM [98]. The Ca^2+^ spark peak was ~100.1 µM (Figure A1), which is in agreement with the estimate that due to the small subspace, the elevation of free calcium can be two orders of magnitude larger than the resting value [99]. The plateau in the spark profile of free calcium explains the energy coupling effect between the closed and open channels, before the Ca^2+^ spark is completely terminated. Similar to the study of [27], the effect of IP_3_R has not been incorporated in this model study. Thus, the involvement of other leak pathways like via IP_3_-receptors (IP3R) has not been excluded, though the functional role of IP_3_R at rest has not been confirmed. As shown in Figure A1D, the higher frequency of [Ca^2+^/CaM] elevation in the quiescent cell model provided the evidence of non-spark events where calcium release via the opening of one or a few RyRs bind to calmodulin buffer, as well as SR and SL buffers.

## Appendix B. Model Equations and Parameters

### Appendix B.1. Calcium Release Site (CRU)

The differential equations describing Ca^2+^ at the release site are as follows:

(A1) $$\frac{d{\left[Ca\right]}_{ds}^{\left(i\right)}}{dt}=\frac{\left(J_{ryr}^{\left(i\right)}-J_{efflux}^{\left(i\right)}+J_{dhpr}^{\left(i\right)}\right)}{\lambda_{ds}}-2\frac{d{\left[CaM\right]}^{\left(i\right)}}{dt}-\frac{d{\left[CaSL\right]}^{\left(i\right)}}{dt}-\frac{d{\left[CaSR\right]}^{\left(i\right)}}{dt}$$

(A2) $$\frac{d{\left[CaM\right]}^{\left(i\right)}}{dt}=k_{CaM}^{+}{\left({\left[Ca\right]}_{ds}^{\left(i\right)}\right)}^{2}\left({\left[CM\right]}_{T}-{\left[CaM\right]}_{ds}^{i}\right)-k_{CM}^{-}{\left[CaM\right]}_{ds}^{i}$$

(A3) $$\frac{d{\left[CaSL\right]}^{\left(i\right)}}{dt}=k_{SL}^{+}\left({\left[Ca\right]}_{ds}^{i}\right)\left({\left[SL\right]}_{T}-{\left[CaSL\right]}_{ds}^{i}\right)-k_{SL}^{-}{\left[CaSL\right]}_{ds}^{i}$$

(A4) $$\frac{d{\left[CaSR\right]}^{\left(i\right)}}{dt}=k_{SR}^{+}\left({\left[Ca\right]}_{ds}^{i}\right)\left({\left[SR\right]}_{T}-{\left[CaSL\right]}_{ds}^{i}\right)-k_{CM}^{-}{\left[CaSR\right]}_{ds}^{i}$$

where (*i*) is an index indicating the *i*th specific CRU, $\lambda_{ds}=V_{ds}/V_{myo}$ is the volume fraction that scale the fluxes, defined based on myoplasmic volume, to the subspace volume compartment. The membrane buffers used here are similar to those used previously [58,100].

### Appendix B.2. Ryanodine Receptor Type-2 Model

The states of the RyR2 are

(A5) $$C\begin{array}{c} \overset{k_{ryr}^{+}}{\to }\\ \underset{k_{ryr}^{-}}{\leftarrow } \end{array}O$$

with the transition rates

(A6) $$k_{ryr}^{+}=12{\left({\left[{\mathrm{Ca}}^{2+}\right]}_{ds}^{i}\right)}^{2}\cdot \left(2.8\times 10^{-4}{\left[{\mathrm{Ca}}^{2+}\right]}_{jsr}^{i}+0.02\right)$$

(A7) $${\left[{\mathrm{Ca}}^{2+}\right]}_{ds}^{i}=500$$

Coupling of the RyR2 channels in the cluster uses allosteric coupling energies: $\varepsilon_{OO},\;\varepsilon_{CC}$ as before [27]. At each release site, the transition between two cluster states Si and Sj is represented as

(A8) $$S_{i}\begin{array}{c} \overset{\chi_{CO}k_{ryr}^{+}N_{C\to O}}{\to }\\ \underset{\chi_{OC}k_{ryrN_{O\to C}}^{-}}{\leftarrow } \end{array}S_{j}$$

where $N_{O\to C}\left(N_{C\to O}\right)$ represents the number of channels in cluster state Si (Sj) that can be switched from Open to Closed (Closed to Open). The coupling gating in the cluster is given by the formula

(A9) $$\chi_{co}=\exp\left(-{\bar{a}}_{j}\times 0.5\times \left(N_{closed}\varepsilon_{cc}-\left(N_{open}-1\right)\varepsilon_{oo}\right)\right)$$

(A10) $$\chi_{oc}=\exp\left(-{\bar{a}}_{j}\times 0.5\times \left(N_{open}\varepsilon_{oo}-\left(N_{closed}-1\right)\varepsilon_{cc}\right)\right)$$

with $\bar{a_{j}}$ is the average allosteric connectivity for each RyR based on mean-field approach [54]. The variables $N_{closed}$ and $\;N_{open}$ represent the current number of non-conducting, and conducting channels in the cluster state Si, respectively.

The non-junctional or ‘rogue’ RyR is modelled deterministically using the formula

(A11) $$J_{ryr-nj}=P_{O,ryr-nj}\cdot \left({\left[{\mathrm{Ca}}^{2+}\right]}_{nsr}-{\left[{\mathrm{Ca}}^{2+}\right]}_{myo}\right)$$

(A12) $$v_{ryr-nj}=v_{ryr}^{T}\times \left[{\mathrm{fraction}}_{\mathrm{nj}-\mathrm{RyR}}\;\right]$$

(A13) $$\frac{dP_{o,ryr-nj}}{dt}=k_{ryr-nj}^{+}\left(1-P_{o,ryr-nj}\right)-k_{ryr-nj}^{-}P_{o,ryr-nj}$$

(A14) $$\frac{dP_{o,ryr-nj}}{dt\;}=k_{ryr-nj}^{+}\left(1-P_{o,ryr-nj}\right)-k_{ryr-nj}^{-}P_{0,ryr-nj}$$

$k_{ryr-nj}^{+}$ has the same functional form as $k_{ryr}^{+}$, except the subspace calcium [Ca^2+^]_ds_ in the formula of is now replaced by [Ca^2+^]_myo_. In the model, the fraction of non-junctional RyRs (ryr-nj) is 5%.

### Appendix B.3. L-Type Ca^2+^ Channel Model

The 6-state L-type Ca^2+^ channel (LCC) model, Figure 2, is derived from 5-state LCC model for rat ventricle myocyte from Sun and co-workers with parameters adjusted for the new spark model [29]. Permeation of the L-type Ca^2+^ channel used the Goldman-Hodgkin-Katz (GHK) formalism was used [101]

(A15) $$I_{dhpr}^{\left(i\right)}=N_{open,\;dhpr}^{\left(i\right)}\frac{P_{dhpr}z_{Ca}^{2}V_{m}}{\frac{RT}{F^{2}}}\frac{{\left[Ca^{2+}\right]}_{ds}^{\left(i\right)}\exp\left(\frac{z_{Ca}V_{m}}{\frac{RT}{F}}\right)-\frac{\beta_{o}}{\beta_{I}\cdot {\left[Ca^{2+}\right]}_{o}}}{\exp\left(\frac{z_{Ca}V_{m}}{\frac{RT}{F}}\right)-1}$$

with (*i*) represents the index of a release site $N_{open,\;dhpr}^{\left(i\right)}$ is the number of open DHPR channels, ${\left[{\mathrm{Ca}}^{2+}\right]}_{ds}^{\left(i\right)}$ is the calcium concentration in a dyadic subspace), ${\left[{\mathrm{Ca}}^{2+}\right]}_{o}$ is the extracellular calcium concentration; P_dhpr_ is the single channel permeability; z_Ca_ = 2 is the valence of Ca^2+^ ion; R is the universal gas constant, T is the temperature and F is the Faraday constant. The partition coefficients are $\beta_{0}=0.341,\;\beta_{1}=1$ in the case of Ca^2+^ channel [102,103]. NOTE: If V_m_ = 0, this formula is used instead

(A16) $$I_{dhpr}^{\left(i\right)}=N_{open,\;dhpr}^{\left(i\right)}P_{dhpr}z_{Ca}F\left({\left[Ca^{2+}\right]}_{ds}^{\left(i\right)}-{\left[Ca^{2+}\right]}_{o}\right)$$

Even though the role of extracellular calcium was not investigated in this study, the model also considered the effect of extracellular calcium [Ca^2+^]_o_ on the model by modulating single channel current and shifting the half-activation voltage V1/2.

(A17) $$V_{\frac{1}{2}}=-40.344+13.31\times \exp\left(-{\left[Ca\right]}_{o}\times \frac{10^{-3}}{0.053}\right)+31.65\;\;\;\;\times \exp\left(-{\left[Ca\right]}_{o}\times \frac{10^{-3}}{11.305}\right)+3$$

It has been suggested that CaM tethers to a region of C-terminal between an EF and IQ domain of LCC [104,105], with 2 different segments (A and C sequences) within the pre-IQ motif of LCC have been indicated to be critical for CDI [106], and this tethering occurs at very low level of calcium concentration (20–100 nM) [35]. This is the range of resting calcium level; thus, it is assumed that all CaM tethers to the channels all the time. So, the total [CaM] is assumed fixed in the subspace, and calcium-dependent inactivation of LCC (k2→4) is modelled via calcium-Calmodulin (CaCaM) complex. In the model, similar to the experimental data, it is assumed that a single bound Ca^2+^/CaM complex is both necessary and sufficient for CDI [107]. Hence, the transition rate for CDI is formulated as k24 = c24 [Ca^2+^/CaM].

Not only Ca^2+^, but also other ions can also permeate via LCC [108,109]. However, due to the large permeability of Ca^2+^ compared to other ions (e.g., PCa/PNa > 1000), in this study, only Ca^2+^ current is modelled. Due to the nonlinearity in the I-V curve and based on the assumption of independent permeation between ion species and constant-field theory, the Goldman-Hodgkin-Katz (GHK) formalism was used [101].

The equations for the LCC model are given in Table A1. The fraction of LCC channels in each state during a +10 (mV) voltage clamp is shown in Figure 2B. State 2 and 3 are open states; while State 4 is calcium-dependent inactivation and State 5 is voltage-dependent inactivation.

### Appendix B.4. Na^+^ Channel Model

The whole-cell Na^+^ current was derived from the formula

(A18) $$I_{Na}=g_{Na}m_{Na}h_{Na}^{3}j_{Na}\left(V_{m}-E_{Na}\right)$$

with the ODE for gating variable follows the first-order differential equation [108]

(A19) $$\frac{dx_{Na}}{dt}=\frac{x_{\infty }-x_{Na}}{\tau_{x}}$$

where *x_Na_* represents the dimensionless gating variables, either *m*, *h*, or *j*, in the range between 0 and 1.0 and $x_{\infty }$ is the steady-state value for the gating variable. The unit for the time constants is in seconds.

(A20) $$h_{\infty }=\frac{1}{1+\exp\left(\frac{V_{m}+76.1}{6.07}\right)}$$

(A21) $$j_{\infty }=h_{\infty }$$

(A22) $$m_{\infty }=\frac{1}{1+\exp\left(\frac{V_{m}+48}{-6.5}\right)}$$

(A23) $$\tau_{m}=\frac{0.00136}{0.32\left(V_{m}+47.13\right)/\left(1-\exp\left(-0.1\left(V_{m}+47.13\right)\right)+0.08\times \exp\left(-\frac{V_{m}}{11}\right)\right)}$$

If V_m_ >= −40

(A24) $$\tau_{h}=0.0004537\left(1+\exp\left(\frac{V_{m}+10.66}{-11.1}\right)\right)$$

(A25) $$\tau_{j}=\frac{0.01163\left(1+\exp\left(-0.1\left(V_{m+32}\right)\right)\right)}{\exp\left(-2.535\times 10^{-7}V_{m}\right)}$$

If V_m_ < −40

(A26) $$\tau_{h}=\frac{0.00349}{0.81\exp\left(\frac{V_{m}+80}{-6.8}\right)+3.56\exp\left(0.079V_{m}\right)+3.1\times 10^{5}\exp\left(0.35V_{m}\right)}$$

(A27) $$\tau_{j}=\frac{0.00349}{\begin{array}{c} \frac{5\left(V_{m}+37.78\right)}{1+\exp\left(0.311\left(V_{m}+79.23\right)\right)}\left(-12,714\exp\left(0.2444V_{m}\right)-3.474\times 10^{-5}\exp\left(-0.04391V_{m}\right)\right)+\\ \frac{0.1212\exp\left(-0.01052V_{m}\right)}{1+\exp\left(-0.1378\left(V_{m}+40.14\right)\right)} \end{array}}$$

(A28) $$h_{\infty }=\frac{0.135\exp\left(\frac{V_{m}+80}{-6.8}\right)}{0.135\exp\left(\frac{V_{m}+80}{-6.8}\right)+3.56\exp\left(0.079V_{m}\right)+3.1\times 10^{5}\exp\left(0.35V_{m}\right)}$$

(A29) $$j_{\infty }=\frac{\frac{V_{m}+37.78}{1+\exp\left(0.311\left(V_{m}+79.23\right)\right)}\left(-12,714\exp\left(0.2444V_{m}\right)-3.474\times 10^{-5}\exp\left(-0.04391V_{m}\right)\right)}{\begin{array}{c} \frac{V_{m}+37.78}{1+\exp\left(0.311\left(V_{m}+79.23\right)\right)}\left(-12,714\exp\left(0.2444V_{m}\right)-3.474\times 10^{-5}\exp\left(-0.04391V_{m}\right)\right)+\\ \frac{0.1212\exp\left(-0.010527V_{m}\right)}{1+\exp\left(-0.1378\left(V_{m}+40.14\right)\right)} \end{array}}$$

### Appendix B.5. K^+^ Channel Models

There are four different K^+^ channel currents (I_tof_, I, I_K1_, and I_Kss_). The formula for fast and slow transient outward currents (I_Ktof_, I_Ktos_, respectively) are based on the model developed using data observed in mouse [49].

(A30) $$I_{K1}=g_{K1}\times \frac{{\left[K\right]}_{o}}{{\left[K\right]}_{o}+210}\times \frac{\left(V_{m}-E_{K}-1.73\right)}{1+\exp\left(0.0896\left(V_{m}-E_{K-1.73}\right)\right)}$$

(A31) $$I_{kss}=g_{Kss}\times a_{Kss}*i_{Kss}\left(V_{m}-E_{K}\right)$$

(A32) $$I_{ksf}=g_{Ktof}\times a_{Ktof}*i_{Ktof}\left(V_{m}-E_{K}\right)$$

(A33) $$I_{ktos}=g_{Ktos}\times a_{Ktos}*i_{Ktos}\left(V_{m}-E_{K}\right)$$

(A34) $$\frac{da_{Kss}}{dt}=10^{3}\frac{\frac{1.0}{1.0+\exp\left(-\frac{V_{m}+22.5}{7.7}\right)}-a_{Kss}}{0.13+0.393\times 0.393\times \exp\left(-0.0862V_{m}\right)}$$

(A35) $$\frac{da_{Ktof}}{dt}=10^{3}\times \left[0.18064\exp\left(\left(V_{m}+30\right)\times 0.3577\right)\times \left(1-a_{Ktof}\right)\;\;\;\;\;\;\;\;\;-0.3956\exp\left(-\left(V_{m}+30\right)\times 0.06237\right)\times a_{Ktof}\right]$$

(A36) $$\frac{da_{Ktof}}{dt}=10^{3}\times [\frac{\left(0.000152\exp\left(-\frac{V_{m}+13.5}{7}\right)\right)}{1+0.067083\exp\left(-\frac{\left(V_{m}+33.5\right)}{7}\right)}\times \left(1-a_{ktof}\right)\;\;\;\;\;\;-\frac{0.00095\exp\left(\frac{V_{m}+33.5}{7}\right)}{1+0.051335\exp\left(\frac{\left(V_{m}+33.5\right)}{7}\right)}\times i_{Ktof}]$$

(A37) $$\frac{da_{Ktos}}{dt}=10^{3}\frac{\frac{1.0}{1.0+\exp\left(-\frac{V_{m}+22.5}{7.7}\right)}-a_{Ktos}}{2.058+0.493\times \exp\left(-0.0629V_{m}\right)}$$

(A38) $$\frac{di_{Ktos}}{dt}=10^{3}\frac{\frac{1.0}{1.0+\exp\left(\frac{V_{m}+45.20}{5.70}\right)}-i_{Ktos}}{270.0+1050/\left(1+\exp\left(\frac{V_{m}+45.2}{5.70}\right)\right)}$$

with $g_{K1}=0.15;g_{Kss}=0.0421;g_{Ktof}=0.0798;g_{Ktos}=0.00629;g_{bK}=1.38\times 10^{-7}$.

The initial values are given in Table A2.

### Appendix B.6. Sarcolemmal Pumps/Exchangers

The two extrusion pathways for calcium via SL are plasma-membrane Ca^2+^/ATP-ase (PMCA) and Na^+^/Ca^2+^ exchanger (NCX).

(A39) $$J_{ncx}=\frac{-A_{m}I_{ncx}}{FV_{myo}}$$

with *A_m_* is the SL surface area and *V_myo_* is the myoplasmic volume

The NCX is given by the formula

(A40) $$I_{\frac{Na^{+}}{K^{+}}}={\bar{I}}_{\frac{Na^{+}}{K^{+}}}\frac{1}{\left(1+0.01245\exp\left(-0.1\left(V_{m}\frac{F}{RT}\right)\right)\times 0.0365\left(\frac{1}{7}\exp\left(\frac{{\left[Na\right]}_{o}}{67,300}\right)-1\right)\exp\left(-V_{m}\frac{F}{RT}\right)\right)}\;\times \frac{1}{1+{\left(\frac{21,000}{{\left[Na\right]}_{i}}\right)}^{1.5}\frac{{\left[K\right]}_{o}}{{\left[K\right]}_{o}+1500}}$$

with ${\bar{I}}_{ncx}$ is the maximal NCX current. The parameters are given in Table A3.

(A41) $$I_{pmca}={\bar{I}}_{pmca}\frac{{\left({\left[{\mathrm{Ca}}^{2+}\right]}_{myo}\right)}^{\eta_{pmca}}}{{\left(K_{m,pmca}\right)}^{\eta_{pmca}}+{\left({\left[{\mathrm{Ca}}^{2+}\right]}_{myo}\right)}^{\eta_{pmca}}}$$

In the case of sodium and potassium pumps, we have

### Appendix B.7. Background Currents

The background currents follow the linear Ohm-law

(A42) $$I_{bX}=g_{X}\left(V_{m}-E_{X}\right)$$

The three different background currents were used in the model

(A43) $$I_{bCa}=g_{bCa}\left(V_{m}-E_{Ca}\right)$$

(A44) $$I_{bNa}=g_{bNa}\left(V_{m}-E_{Na}\right)$$

(A45) $$I_{bK}=g_{bK}\left(V_{m}-E_{K}\right)$$

with the reversal potentials are derived from Nernst equation.

(A46) $$E_{X}=\frac{RT}{Fz_{X}}\ln\left(\frac{{\left[X\right]}_{i}}{{\left[X\right]}_{o}}\right)$$

with *z_x_* is the valance of ion *X*.

### Appendix B.8. Sarcoplasmic Reticulum Ion Pumps

The 2-state reduced model of the Tran-Crampin model was chosen with parameters selected so that the maximum pumping rate per pump molecule, max (*v_serca_*), is 5 s^−1^.

(A47) $$J_{serca}=2\times v_{serca}$$

(A48) $$v_{serca}=\frac{3.24873\times 10^{12}{\left(K_{myo}\right)}^{2}+K_{myo}\left(9.17846\times 10^{6}-11,478.2K_{sr}\right)-0.329904K_{sr}}{D_{cycle}}$$

(A49) $$D_{cycle}=0.104217+17.923K_{sr}+K_{myo}\left(1.75583\times 10^{6}+7.61673K_{sr}\right)+\;{\left(K_{myo}\right)}^{2}\left(6.08463\times 10^{11}+4.50544\times 10^{11}K_{sr}\right)$$

and

(A50) $$K_{myo}={\left(\frac{{\left[Ca\right]}_{myo}}{10^{-3}K_{d,myo}}\right)}^{2};\;K_{sr}={\left(\frac{{\left[Ca\right]}_{nsr}}{10^{-3}K_{d,sr}}\right)}^{2}$$

### Appendix B.9. Sarcoplasmic Reticulum Ion Pumps

The three endogenous buffers of calmodulin (CaM), troponin (Trpn), and the phospholipids of the SR membrane (SRbuf) are used for the bulk myoplasm. The buffering parameters are listed in Table A4.

(A51) $$\frac{d\left[Ca;CaM\right]}{dt}=k_{CM}^{+}\left({\left[Ca\right]}_{myo}^{2}\right)\left({\left[CM\right]}_{T}-{\left[Ca;CaM\right]}_{myo}\right)-k_{CM}^{-}{\left[Ca;CaM\right]}_{myo}$$

(A52) $$\frac{d\left[Ca;SRbuf\right]}{dt}=k_{SR}^{+}\left({\left[Ca\right]}_{myo}\right)\left({\left[SRbuf\right]}_{T}-{\left[Ca;SRbuf\right]}_{myo}\right)\;-k_{SR}^{-}{\left[Ca;SRbuf\right]}_{myo}\;\;$$

(A53) $$\frac{d\left[Ca;Trpn\right]}{dt}=k_{Trpn}^{+}\left({\left[Ca\right]}_{myo}\right)\left({\left[Trpn\right]}_{T}-{\left[Ca;Trpn\right]}_{myo}\right)\;-k_{Trpn}^{-}{\left[Ca;Trpn\right]}_{myo}\;\;$$

### Appendix B.10. Membrane Potential

During AP, the dynamics of the membrane voltage V_m_ is governed by the equation

(A54) $$\frac{dV_{m}}{dt}=-\frac{10^{3}}{C_{sc}}\left(\begin{array}{c} \frac{\left(I_{dhpr}^{T}\right)}{A_{m}}+I_{Na}+I_{NCX}+I_{NaK}+I_{pmca}+I_{K1}+I_{Kss}+I_{Ktof}+\\ I_{Ktos}+I_{background}+I_{app} \end{array}\right)$$

with NSFU is the number of Ca^2+^ release sites, and

(A55) $$I_{dhpr}^{T}=\overset{N_{SFU}}{\underset{i=1}{\sum }}I_{dhpr}^{i}$$

(A56) $$I_{background}=I_{bCa}+I_{bNa}+I_{bK}$$

NOTE: The units: membrane potential and reversal potential are in mV, while other parameters are given in the form of unit-density, e.g., the specific membrane capacitance C_sc_ = 1 µF/cm^2^, the ionic currents are given in (µA/cm^2^), except the L-type currents are measured at whole-cell level (µA) which needs to be converted to current density by dividing the membrane surface area A_m_ (cm^2^).

### Appendix B.11. Parameters

The rat ventricular cell volume was chosen 25 pL to match to the number 20,000 CRUs per cell with Cm/Vcell = 7.12 pF/pL [99,100]. The schematic diagram of the compartmental model of the rat ventricular myocyte with all the ionic currents is given in Figure A1.

**Table A1:** Parameters for L-type Ca^2+^ channel model.

$P_{o2}=\frac{-1}{1+e^{\frac{v-v_{\frac{1}{2}}}{S}}}+1$
$V_{\frac{1}{2}}=-40.344+13.31\times e^{\left(-\frac{{\left[Ca\right]}_{o}}{53.0}\right)}+31.65\times e^{\left(-\frac{{\left[Ca\right]}_{o}}{11,305}\right)}+3$
$S=\left(0.632+0.368\times e^{\left(\frac{-{\left[Ca\right]}_{0}}{10^{3}}-0.1\right)/0.784)}\right)\times \left(-\frac{3.792}{1+e^{\left(\frac{{\left[Ca\right]}_{0}}{10^{3}}-0.0469\right)/0.011)}+6.39}\right)$
$att=130+\frac{-129}{1+e^{\frac{V_{m}-60}{8}}}+\frac{0.76}{1+e^{\frac{V_{m}-5}{3.14}}}$
$\tau_{P_{02}}=0.00025+0.00305\times e^{-0.0045{\left(V_{m}+7\right)}^{2}}+0.00105\times e^{-0.002{\left(V_{m}-18\right)}^{2}}$
$inch_{Ca}=0.89+\frac{-0.888}{1+e^{\frac{V_{m}-43.066}{2.289}}}\times \left(1.004+\frac{-1.199}{1+e^{\frac{\frac{{\left[Ca\right]}_{o}}{10^{3}}-0.259}{0.164}}}\right)$
$k_{1,5}=\frac{k_{5,1}k_{1,2}k_{2,5}}{k_{5,2}k_{2,1}}$
$k_{5,4}=\frac{k_{4,5}k_{5,2}k_{2,4}}{k_{4,2}k_{2,5}}$
$d_{dhpr}=d_{dhpr,\left({\left[Ca\right]}_{0}=1.8\mathrm{mM}\right)}\left(1+\frac{-1}{1+e^{\frac{\frac{{\left[Ca\right]}_{o}}{10^{3}}-0.08}{0.0159}}}\right)$
$k_{1,2}=0.115\times \frac{P_{O2}}{\tau_{O2}}$
$k_{2,1}=0.115\times \frac{1-0.155\times P_{O2}}{\tau_{O2}}\times att$
$k_{2,3}=0.7\times (987.3+\frac{-903.53}{1+e^{\frac{V_{m}-51.23}{6.785}}}$)
$k_{3,2}=500$
$k_{2,5}=0.5\times \left(32+\frac{-18.46}{1+e^{\frac{V_{m}-30}{5.746}}}\right)\times (1-inh_{Ca})$
$k_{5,2}=1.0\times \left(32+\frac{-5.03}{1+e^{(V_{m}-30)/0.787}}\right)+\left(0.95+\frac{-0.93}{1+e^{\frac{V_{m}+40}{0.787}}}\right)$
$k_{2,4}=8\times \left[CaM\right]$
$k_{4,2}=1$
$k_{5,1}=6.9$
$k_{4,5}=600$
$k_{3,2}=500$
$k_{6,1}=1\times (0.0022+\frac{2998.978}{1+e^{-\left(Vm+45\right)\times 0.71428}})$
$k_{1,6}=1\times (3000+\frac{2998.978}{1+e^{-\left(Vm+45\right)\times 0.6667}})$

**Table A2:** Initial values.

Variables	Value	Unit
m_Na_	0.0015d0	Unitless
h_Na_	0.9849d0	Unitless
j_Na_	0.9849d0	Unitless
a_Kss_	0.0021d0	Unitless
in_Kss_	1.d0	Unitless
a_Ktof_	0.0021d0	Unitless
in_Ktof_	1.d0	Unitless
a_Ktos_	2.9871d-04	Unitless
in_Ktos_	0.9994d0	Unitless
[Ca^2+^]_myo_	0.08769	µM
[Ca^2+^]_nsr_	1.00737d3	µM
[Na^+^]_i_	1.02d4	µM
[K^+^]_i_	1.4372d5	µM

**Table A3:** Constant data and whole-cell level data.

Variables	Value	Unit
Faraday constant (F)	9.6485d4	C/mol
Universal gas constant (R)	8.314d3	mJ/(mol.K)
Temperature (T)	310	K
[Na]_o_	1.4d5	µM
[Ca]_o_	1.8d3	µM
[K]_o_	5.4d3	µM
Cell volume (V_cell_)	25.0	pL
Myoplasmic volume (V_myo_)	12.5	pL
Network SR volume ($V_{nsr}^{}$)	0.762d0	pL
Junctional SR volume ($V_{jsr}^{T}$)	1.25d-1	pL
Subspace volume ($V_{ds}^{T}$)	1.55d-2	pL
Concentration [SERCA] K_d,myo_ K_d,sr_	300 900 2150	µM µM µM
RyR release rate ($v_{ryr}^{T}$)	56.4279	1/s
Percent nj-RyR	0.5	Unitless
Transfer rate from subspace to bulk myoplasm ($v_{efflux}^{T}$)	300	1/s
Refill rate from nSR to jSR ($v_{refill}^{T}$)	2.4	1/s
NCX maximum current density ($\bar{I_{ncx}}$) K_d,ncxNa_ K_d,ncxCa_	520 8750 1380	µA/µF
PMCA maximum current density ($\bar{I_{pmca}}$) K_d,pmca_	0.12 0.5	µA/µF
Na/K maximum current density ($\bar{I_{Na/K}}$)	0.88	µA/µF

**Table A4:** Calcium buffers.

Variables	Value	Unit
General buffer in myoplasm K_d_	1.23d2 0.96	µM µM
General buffer in jSR (e.g., Calsequestrin) Kd	1.4d4 6.38d2	µM µM
High-affinity Troponin C $k_{CM}^{+}$ $k_{CM}^{-}$	1.40d2 2.37 3.2d-2	µM 1/(µM.s) 1/s
Calmodulin $k_{SR}^{+}$ $k_{SR}^{-}$	2.4d1 3.0d1 7.14d1	µM 1/(µM.s) 1/s
SL buffer $k_{SL}^{+}$ $k_{L}^{-}$	250 115 1000	µM 1/(µM.s) 1/s
SR buffer $k_{SR}^{+}$ $k_{SR}^{-}$	47 115 100	µM 1/(µM.s) 1/s
